# Clinical characteristics and impact of comorbidities on the prognosis of senile epilepsy in Southwest China: a retrospective cohort study

**DOI:** 10.1186/s42494-024-00153-8

**Published:** 2024-04-01

**Authors:** Zhen Cao, Yinping Li, Shengyi Liu, Zihua He, Jinmei Li

**Affiliations:** 1 Department of Neurology, West China Hospital, Sichuan University, Chengdu, 610041 China; 2 Department of Geriatric, The Second People’s Hospital of Yibin, Yibin, 644000 China; 3 Department of Neurology, Chengdu Shangjin Nanfu Hospital, Chengdu, 610000 China

**Keywords:** Senile epilepsy, Elderly, Epilepsy, Comorbidity, Prognosis

## Abstract

**Background:**

Senile epilepsy and its comorbidities pose a tremendous burden on patients and the society. This study was aimed to investigate the clinical characteristics and comorbidities of senile epilepsy, as well as the impact of comorbidities on the prognosis of senile epilepsy.

**Methods:**

Information of patients with senile epilepsy was retrospectively collected from three tertiary hospitals in Southwest China between December 2014 and December 2022. A total of 154 patients met the inclusion criteria and were divided into two groups based on the presence or absence of comorbidities. The prevalence, type, characteristics, and impacts of the comorbidities were investigated. The characteristics of patients with and without comorbidities were also compared.

**Results:**

Eighty-one percent of patients with senile epilepsy had at least one comorbidity, and 36% had three or more comorbidities. Eighteen different types of comorbidities were identified. The most common comorbidities were neurological (61%), followed by cardiovascular (45%) and psychiatric (26%) comorbidities. More than one-third of patients had bidirectional comorbidities, whereas more than half of the patients had additional causal comorbidities. Among all types of comorbidities, neurological and psychiatric comorbidities were found to be associated with an increased risk of recurrent seizures. Compared to patients without bidirectional comorbidities, those with at least one bidirectional comorbidity had a lower rate of achieving seizure freedom. The higher the number of bidirectional comorbidities, the lower the seizure-free rate. Survival analysis revealed that patients with neurological comorbidities had a higher risk of death.

**Conclusions:**

This study revealed a high comorbidity rate and a low seizure-freedom rate among patients with senile epilepsy. In particular, neuropsychiatric comorbidities can increase the risk of seizures and affect the survival rate of patients with senile epilepsy. Therefore, preventing and managing these comorbidities may improve seizure outcomes and reduce mortality in this special population.

## Background

Epilepsy is a chronic neurological disorder that can affect people of all ages. According to the Global Burden of Diseases report, epilepsy is the third most common neurological condition, affecting an estimated number of 65 million people globally. The prevalence of epilepsy is approximately 6 per 1000 with an annual incidence of ~ 68 per 100,000 persons [1]. Senile epilepsy occurs after the age of 60 or 65 [2]. Although the incidence and prevalence of epilepsy greatly vary across different countries and regions with varying socioeconomic and cultural backgrounds, they are generally higher in the elderly individuals aged over 60 or 65 years than other age groups [3]. The current studies suggest that the annual incidence of epilepsy in the elderly is approximately 100–134 per 100,000 [3, 4], which is nearly twice as high as that in the younger population [3], and the annual prevalence is approximately 1–5.4% [4, 5]. Moreover, the incidence and prevalence of epilepsy among the elderly will grow even higher with the increase of the elderly population. Thus, epilepsy has emerged as the third most prevalent disease of the nervous system in the elderly, only after cerebrovascular disease and Alzheimer’s disease [6].

Comorbidity refers to the simultaneous occurrence of two or more medical conditions in the same individual [7]. Patients with epilepsy may have one or more comorbidities, such as physical, neurological, and mental conditions [8–10]. The prevalence of comorbidities in patients with epilepsy is significantly higher than that in the general population and in patients with other chronic diseases. Studies have suggested that there may be common pathophysiological mechanisms between epilepsy and its comorbidities [8–10]. Comorbidities with causal effects can significantly impact the prognosis of epilepsy such as post-stroke epilepsy, which account for about 10% [10]. This is followed by bidirectional comorbidities, in which epilepsy and other conditions affect each other but with no causal relationship, such as anxiety and depression. Therefore, comorbidities are increasingly recognized as an important factor affecting the prognosis and quality of life of epilepsy patients [9].

Although the number of elderly epilepsy patients is increasing, there is still a large gap in the prevalence of comorbidities in patients with senile epilepsy and the impact of the comorbidities on the prognosis of senile epilepsy [11]. Therefore, this retrospective study was conducted to investigate the relationship between senile epilepsy and its comorbidities in Southwest China and the impact of comorbidities on prognosis, including seizure outcomes and survival.

## Methods

### Sources of study data

Information of senile epilepsy patients was collected from the outpatient and inpatient systems in West China Hospital of Sichuan University, the Second People’s Hospital of Yibin, and Chengdu Shangjin Nanfu Hospital from December 2014 to December 2022. The inclusion criteria were epilepsy onset after the age of 60, availability of reliable and complete data on comorbidities, and treatment duration of more than one year. All the relevant data were collected from the medical record systems and through in-person or telephone interviews with the patients.

### Measurement

#### General information

General information included sex, age, educational level, and place of residence. The levels of education were classified as illiterate, primary school level, junior high school level, senior high school level, and college level.

#### Clinical characteristics of epilepsy

The clinical characteristics of epilepsy included the age of onset, age of diagnosis, duration of epilepsy, seizure frequency, etiology of epilepsy, seizure type, number of anti-seizure medications (ASMs), neuroimaging, and electroencephalogram (EEG). The etiologies of epilepsy and types of seizure were categorized according to the recent recommendations of the International League Against Epilepsy (ILAE 2017) [12].

#### Comorbidities

Information on comorbidities was obtained by reviewing the medical history and records, in accordance with the International Classification of Diseases, 10th Revision (ICD-10 codes). Depression, anxiety, dementia, and psychotic disorders were classified as bidirectional comorbidities of epilepsy [9]. Cerebral infarction, traumatic brain injury, cerebral hemorrhage, intracranial tumor, and cerebrovascular malformation were classified as causal comorbidities. Comorbidities not of the above two categories were classified as other comorbidities. The comorbidities documented in this study occurred before the onset of senile epilepsy.

#### Outcomes

The seizure control status of patients was categorized as “seizure-free,” “treatment failure,” or “uncertain” according to the ILAE 2010 treatment consensus [13]. Seizure-free was defined as being free from seizures for a minimum period of three times the longest inter-seizure interval before intervention (determined from seizures occurring within the past 12 months) or 12 months, whichever was longer. Treatment failure was defined as the recurrence of seizures despite a proper and adequate intervention; otherwise, the outcome was designated as uncertain [13]. Seizure outcomes in this study were classified as either seizure-free or seizure-uncontrolled (indicating treatment failure or uncertainty).

### Statistical analysis

For statistical analysis, we used the Chinese version of IBM SPSS Statistics 27. A statistically significant result had a two-tailed significance level of *P* ≤ 0.05. Qualitative data are presented as ratios or rates, while quantitative data are presented as the mean ± standard deviation. Patients were categorized into the comorbidity group and the non-comorbidity group. Differences between the two groups were analyzed with the *t/t’* test (when the variance was uneven, the *t’* test was used) or the chi-square test/Fisher’s exact test. Spearman rank correlation test was used to analyze the association between ordinal variables and patient outcomes. Univariate logistic regression was performed on the above variables to calculate the crude odds ratio (OR). Thereafter, general information and epilepsy characteristics were included in the model, and the variable selection method was "forward: conditional". In this process, variables with *P* < 0.05 were included in the model, while those with *P* > 0.10 were excluded. The adjusted OR was subsequently obtained. Survival analysis was conducted using the Kaplan–Meier method. The survival time was defined as the total duration of senile epilepsy, whereas death was identified as the primary outcome. Crude hazard ratios were calculated using Cox regression analysis.

## Results

### Characteristics of senile epilepsy

One hundred and fifty-four patients with senile epilepsy were enrolled in this study. The general data and clinical characteristics of these patients are presented in Table 1. There were no significant differences in sex, age, education, or place of residence between senile epilepsy patients with and without comorbidities. There was also no significant difference in age of onset, age of diagnosis, duration of disease, and number of ASMs. However, the patients with comorbidities were more likely to have focal seizures, structural lesions, and abnormal neuroimaging and EEG features. In terms of seizure outcome, 45% (69/154) of the included patients were seizure-free.

**Table 1:** Comparison of the general information and clinical characteristics of elder patients with and without comorbidities

		**Patients without comorbidity**	**Patients with comorbidities**	**Overall**	***χ***^**2**^***/ t***	***v***	***P*-value**
		***n* (%) / Mean ± SD**	***n* (%) / Mean ± SD**	***n* (%) / Mean ± SD**			
**General information**	**Sex**						
	Female	6 (20.7%)	46 (36.8%)	52 (33.8%)	2.73	1	0.098
	Male	23 (79.3%)	79 (63.2%)	102 (66.2%)			
	**Age (years)**	70.76 ± 5.75	71.54 ± 6.55	71.40 ± 6.40	-0.59	152	0.553
	**Education**						
	Illiteracy	3 (10.3%)	27 (21.6%)	30 (19.5%)	Fisher's exact test		0.443
	Primary school	11 (37.9%)	37 (29.6%)	48 (31.2%)			
	Junior high school	8 (27.6%)	41 (32.8%)	49 (31.8%)			
	Senior high school	2 (6.9%)	8 (6.4%)	10 (6.5%)			
	University	5 (17.2%)	12 (9.6%)	17 (11.0%)			
	**Residence**						
	Rural areas	9 (31.0%)	49 (39.2%)	58 (37.7%)	0.668	1	0.414
	Towns and cities	20 (69.0%)	76 (60.8%)	96 (62.3%)			
**Characteristics of epilepsy**	**Age of onset**	66.17 ± 5.53	67.54 ± 6.40	67.28 ± 6.25	-1.06	152	0.290
	**Age at diagnosis**	67.00 ± 5.47	68.20 ± 6.43	67.98 ± 6.27	-0.93	152	0.354
	**Duration before diagnosis (months)**	9.88 ± 11.76	7.95 ± 7.61	8.31 ± 8.53	1.10	152	0.274
	**Duration of disease (years)**	4.59 ± 3.53	4.00 ± 3.36	4.11 ± 3.39	0.83	152	0.406
	**Seizure frequency before diagnosis (times/month)**	0.68 ± 0.90	0.87 ± 1.22	0.84 ± 1.17	-0.81	152	0.418
	**Number of seizures before diagnosis**	6.93 ± 7.92	5.95 ± 6.16	6.14 ± 6.51	0.73	152	0.467
	**Seizure type**						
	Unknown	6 (20.7%)	11 (8.8%)	17 (11.0%)	Fisher's exact test		0.029*
	Generalized	4 (13.8%)	7 (5.6%)	11 (7.1%)			
	Focal	19 (65.5%)	107 (85.6%)	126 (81.8%)			
	**Etiology**						
	Unknown	23 (79.3%)	25 (20.0%)	48 (31.2%)	Fisher's exact test		< 0.001***
	Metabolic	0 (0.0%)	4 (3.2%)	4 (2.6%)			
	Infectious	1 (3.4%)	0 (0.0%)	1 (.6%)			
	Structural	5 (17.2%)	96 (76.8%)	101 (65.6%)			
	**Number of ASM**						
	0	1 (3.4%)	2 (1.6%)	3 (1.9%)	Fisher's exact test		0.294
	1	26 (89.7%)	98 (78.4%)	124 (80.5%)			
	2	2 (6.9%)	24 (19.2%)	26 (16.9%)			
	3	0 (0.0%)	1 (0.8%)	1 (.6%)			
	**Neuroimaging**						
	Normal	16 (55.2%)	16 (12.8%)	32 (20.8%)	28.43	2	< 0.001***
	Non-specific	6 (20.7%)	22 (17.6%)	28 (18.2%)			
	Abnormal	7 (24.1%)	87 (69.6%)	94 (61.0%)			
	**EEG**						
	Normal	9 (31.0%)	14 (11.2%)	23 (14.9%)	Fisher's exact test		0.027*
	Non-epileptiform	4 (13.8%)	33 (26.4%)	37 (24.0%)			
	Epileptiform	16 (55.2%)	78 (62.4%)	94 (61.0%)			

^*^. *P* < 0.05; ***. *P* < 0.001

### Comorbidities of senile epilepsy

**Fig. 1:**
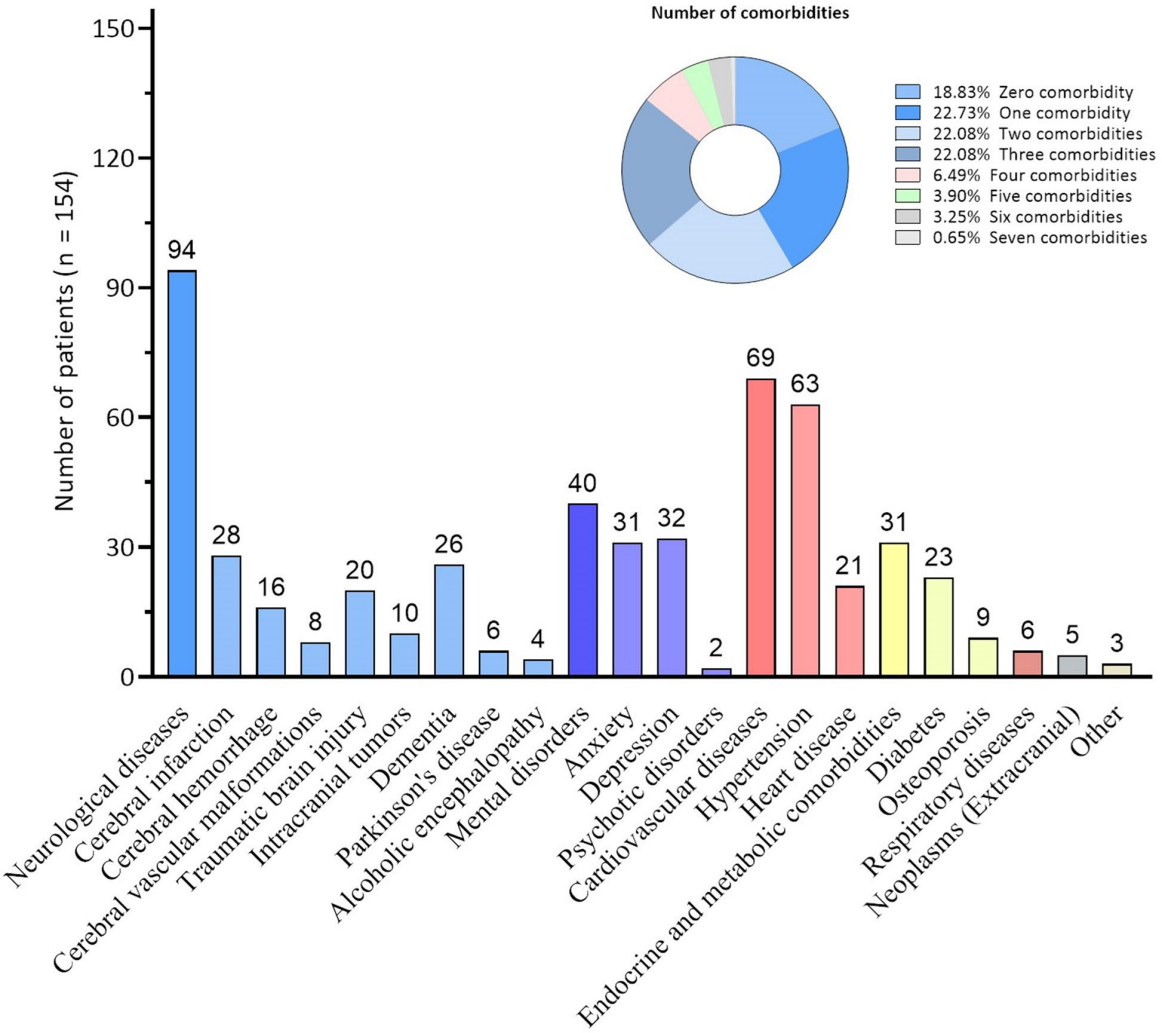
Number of patients with various comorbidities

One hundred and twenty-five patients had at least one comorbidity. As shown in Fig. 1, the total number of comorbidities in each patient ranged 1–7. Specifically, 35 (23%) patients had one comorbidity, 34 (22%) had two comorbidities, 34 (22%) had three comorbidities, and 22 (14%) had more than three comorbidities. A total of 18 different types of comorbidities were identified. The most common comorbidities were neurological (61%), followed by cardiovascular (45%), psychiatric/mental (26%), endocrine and metabolic (20%), respiratory (4%), neoplasms (extracranial) (3%), and other diseases (2%). Of the neurological diseases, cerebrovascular diseases were the most common, affecting 52 of 154 cases.

In addition, 36% of the patients in the comorbidity group had bidirectional comorbidities including depression, anxiety, dementia, and psychotic disorders. More specifically, 18% had one, 14% had two, and 4% had three bidirectional comorbidities. The causal comorbidities included cerebral infarction, traumatic brain injury, cerebral hemorrhage, intracranial tumor, and cerebrovascular malformation. Eighty-one (52%) had at least one causal comorbidity, including one patient with two causal comorbidities and 80 patients with only one causal comorbidity.

### Effect of comorbidities on the seizure-freedom rate

There was a significant difference in the seizure-freedom rate between patients with and without comorbidities (Table 2). However, the significance diminished after adjusting for the confounding factors.

**Table 2:** Comorbidities in elder patients with and without seizure-freedom

	**Seizure-freedom**		**Without seizure-freedom**		***χ***^***2***^	***v***	***P*-value**	**crude OR**	**95% CI**	***P*-value**	**adj. OR**	**95% CI**	***P*-value**
	***n***	**%**	***n***	**%**									
**At least one comorbidity**	51	40.8%	74	59.2%	4.306	1	0.038*	2.374	1.035–5.448	0.041*	2.104	0.880–5.032	0.094
**Number of comorbidities**													
0	18	62.1%	11	37.9%	5.748	3	0.125	1.405	1.054–1.872	0.020*	1.336	0.987–1.808	0.061
1	17	48.6%	18	51.4%									
2	14	41.2%	20	58.8%									
≥ 3	20	35.7%	36	64.3%									
**Comorbidity type**													
**Neurological**	35	37.2%	59	62.8%	5.591	1	0.018*	2.204	1.139–4.265	0.019*	1.705	0.844–3.443	0.137
Traumatic brain injury	7	35.0%	13	65.0%	0.894	1	0.345	1.599	0.600–4.259	0.348	1.441	0.520–3.997	0.482
Intracranial tumors	3	30.0%	7	70.0%	0.416	1	0.519	1.974	0491–7.940	0.338	1.913	0.434–8.443	0.392
Dementia	10	38.5%	16	61.5%	0.509	1	0.476	1.368	0.577–3.243	0.477	1.379	0.558–3.410	0.487
Parkinson's Disease	2	33.3%	4	66.7%	0.025	1	0.875	1.654	0.294–9.312	0.568	3.997	0.609–26.240	0.149
Alcoholic encephalopathy	0	0.0%	4	100.0%	1.733	1	0.188	Ne	-		Ne	-	-
Cerebrovascular disease	23	44.2%	29	55.8%	0.010	1	0.918	1.036	0.529–2.208	0.918	0.824	0.395–1.718	0.605
Cerebral infarction	9	32.1%	19	67.9%	2.219	1	0.136	1.919	0.807–4.566	0.140	1.635	0.654–4.086	0.292
Cerebral hemorrhage	9	56.3%	7	43.8%	0.946	1	0.331	0.598	0.211–1.699	0.335	0.404	0.123–1.324	0.135
Cerebral vascular malformations	5	62.5%	3	37.5%	0.447	1	0.504	0.468	0.108–2.033	0.311	0.462	0.088–2.415	0.360
**Psychiatric**	10	25.0%	30	75.0%	8.571	1	0.003**	3.218	1.440–7.194	0.004**	2.606	1.124–6.041	0.026*
Anxiety	8	25.8%	23	74.2%	5.665	1	0.017*	2.829	1.175–6.811	0.020*	2.665	1.067–6.654	0.036*
Depression	8	25.0%	24	75.0%	6.407	1	0.016*	3.000	1.250–7.199	0.014*	2.469	0.989–6.165	0.053
Psychotic disorders	0	0.0%	2	100.0%	Fisher’s exact test		0.502	Ne	-	-	Ne	-	-
**Cardiovascular**	32	46.4%	37	53.6%	0.125	1	0.724	0.891	0.471–1.688	0.724	0.913	0.461–1.809	0.794
Hypertension	31	49.2%	32	50.8%	0.835	1	0.361	0.740	0.388–1.412	0.361	0.736	0.368–1.473	0.386
Heart disease	7	33.3%	14	66.7%	1.294	1	0.255	1.746	0.663–4.603	0.259	1.677	0.602–4.670	0.322
**Endocrine/metabolic**	10	32.3%	21	67.7%	2.471	1	0.116	1.936	0.842–4.449	0.120	1.953	0.816–4.672	0.133
Diabetes	6	26.1%	17	73.9%	3.831	1	0.050	2.625	0.974–7.077	0.057	2.516	0.890–7.114	0.082
Osteoporosis	5	55.6%	4	44.4%	0.104	1	0.504	0.632	0.163–2.451	0.507	0.767	0.190–3.103	0.710
**Respiratory**	2	33.3%	4	66.7%	0.025	1	0.875	1.654	0.294–9.312	0.568	1.759	0.279–11.080	0.547
**Neoplasms (Extracranial)**	2	40.0%	3	60.0%	0.000	1	1.000	1.226	0.199–7.550	0.826	1.271	0.178–9.097	0.811
**Others**	1	33.3%	2	66.7%	0.000	1	1.000	1.639	0.145–18.460	0.689	2.577	0.225–29.471	0.446
**At least one bidirectional comorbidity**	17	30.9%	38	69.1%	6.680	1	0.010*	2.473	1.235–4.954	0.011*	2.162	1.037–4.506	0.040*
**Number of bidirectional comorbidities**													
0	51	52.0%	47	48.0.%	Fisher’s exact test		0.026*	1.713	1.139–2.577	0.010*	1.578	1.034–2.408	0.034*
1	12	42.9%	16	57.1%									
2	4	18.2%	18	81.8%									
3	2	33.3%	4	66.7%									
**At least one causal comorbidity**	32	39.50%	49	60.50%	1.94	1	0.164	1.574	0.830–2.983	0.165	1.265	0.632–2.532	0.506
**Number of causal comorbidities**													
0	37	50.70%	36	49.30%	Fisher’s exact test		0.125	1.457	0.780–2.721	0.237	1.157	0.588–2.278	0.673
1	31	38.80%	49	61.30%									
2	1	100.00%	0	0.00%									
**At least one other comorbidity**	36	49.40%	53	59.60%	1.63	1	0.203	1.518	0.797–2.893	0.204	1.580	0.798–3.164	0.188
**Number of other comorbidities**													
0	33	50.80%	32	49.20%	2.25	3	0.522	1.254	0.892–1.761	0.192	1.293	0.900–1.857	0.164
1	21	41.20%	30	58.80%									
2	11	44.00%	14	56.00%									
3 +	4	30.80%	9	69.20%									

^*^. *P* < 0.05; **. *P* < 0.01

The rate of seizure freedom was significantly lower in patients with epilepsy who had neurological or psychiatric comorbidities compared to those with other comorbidities. The presence of neurological comorbidity increased the risk of seizures by 120.4% (crude OR [95%] = 2.204 (1.139, 4.265); adjusted OR [95%] = 1.705 [0.844, 3.443]), while the comorbid mental disorders increased the risk of seizures by 221.8% (crude OR [95%] = 3.218 [1.440, 7.194]; adjusted OR [95%] = 2.606 [1.124, 6.041]). The patients with other comorbidities did not show significant difference in the seizure freedom rate from those without comorbidities.

Patients with at least one bidirectional comorbidity had a lower seizure-freedom rate compared to those without bidirectional comorbidities. Thus, the higher the rate of bidirectional comorbidities, the lower the probability of seizure-freedom. Patients with bidirectional comorbidity had a nearly 116% higher risk of seizure than those without bidirectional comorbidities (crude OR [95% CI] = 2.473 [1.235, 4.954]; adjusted OR [95% CI] = 2.162 [1.037, 4.506]). Furthermore, each addition of one bidirectional comorbidity was associated with a 58% increased risk of exacerbating the condition (crude OR [95% CI] = 1.713 [1.139, 2.577]; adjusted OR [95% CI] = 1.578 [1.034, 2.408]).

In terms of the causal comorbidities, there was no statistical difference in the rate of seizure-free outcomes between those with and without at least one causal comorbidity.

### Effect of comorbidities on survival

During the follow-up, 10 out of the 154 (6%) patients died. Three patients died from prolonged status epilepticus, whereas seven died from other causes, including alcoholic cirrhosis, severe pneumonia (*n* = 2), lung cancer, esophageal cancer, cerebral infarction, and heart failure.

**Fig. 2:**
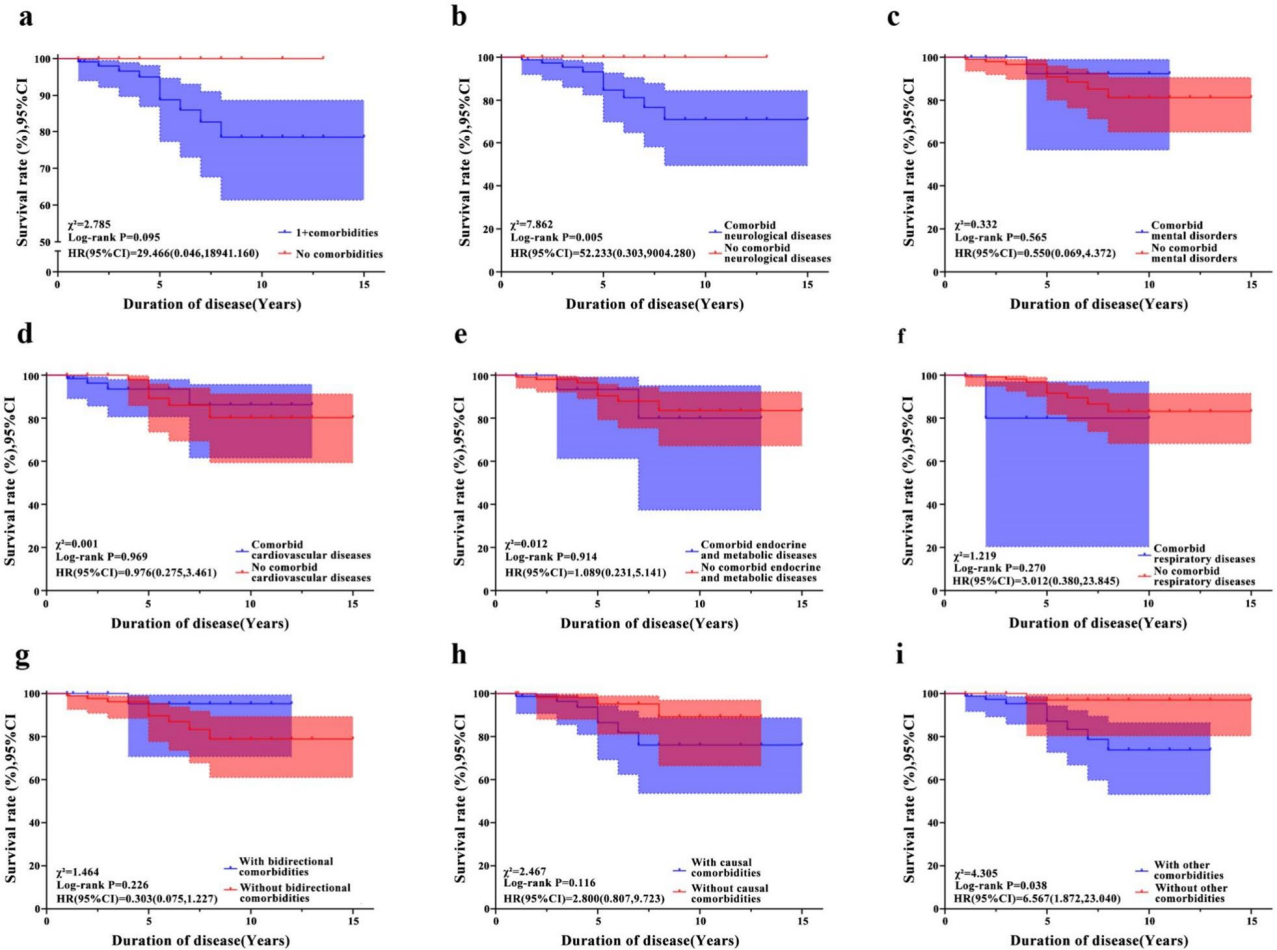
Survival curve analysis showing different survival rates in patients with versus without comorbidities. **a** Patients with comorbidities had a lower survival rate than those without any comorbidity. **b** A lower survival rate was observed in patients with comorbid neurological diseases than those without. **c**-**f** The survival rate did not differ between patients with other system diseases and those without. **g** The survival rate did not differ between patients with and without bidirectional comorbidities. **h** The survival rate did not differ between patients with and without causal comorbidities. **i** The survival rate was lower in patients with other comorbidities than in those without other comorbidities

As shown in Fig. 2a, there was no death among patients without comorbidity, while 10 of 125 (8%) patients in the comorbidity group died. However, survival analysis did not show a statistical significance.

There was no significant difference in the survival rate between patients with a specific type of comorbidity and those without that type (Fig. 2b–f), except that those with comorbid neurological conditions had lower survival rate than those without comorbid neurological conditions.

In addition, there was no significant difference in the survival rate between patients with and without bidirectional comorbidities (Fig. 2g). Similarly, there was no statistically significant difference in survival between those with and without causal comorbidities (Fig. 2h). However, there was a significant difference in survival between patients with and without other comorbidities (Fig. 2i).

## Discussion

The elderly is a distinct population, alongside children and pregnant women. A series of health problems can occur among the elderly due to various physiological changes during the aging process. The coexistence of aging and epilepsy inevitably leads to a prognosis that differs from that in other age groups. Elderly patients often have a large number of underlying diseases. In this retrospective cohort study, we investigated the current status of senile epilepsy and its comorbidities in China and also explored the impact of the comorbidities on the prognosis of senile epilepsy.

Our study found that the main seizure type among people with senile epilepsy was focal seizures, which is consistent with previous studies [2, 14–17]. The most prevalent cause of senile epilepsy was structural lesions, particularly brain abnormalities caused by cerebrovascular diseases such as stroke, as previously reported by other studies [18, 19]. Although the exact etiology of epilepsy is unknown, stroke is the most commonly reported cause of epilepsy, accounting for 30–50% of cases, followed by degenerative diseases and tumors [3–5, 20, 21]. Consistently, in this study, the main cause of senile epilepsy is still structural abnormalities, particularly structural lesions caused by cerebrovascular diseases.

In our study, the seizure-freedom rate among patients with senile epilepsy was only 45%, which was lower than the rate in other populations (70%). This may be because the study population mostly came from rural areas and had lower educational levels, which are often associated with poor health awareness, lower compliance, and thus worse treatment outcomes. In addition, compared with younger patients, older patients with epilepsy have more structural etiologies, which may also have contributed to the lower seizure-freedom rate.

Furthermore, our study found that senile epilepsy patients had more comorbidities compared with epilepsy patients from other age groups [8]. The types of comorbidities were also different from those of other age groups. In population-based studies, the prevalence of psychiatric comorbidities is three times higher among people with epilepsy compared with others over the lifetime. Mood disorders, such as anxiety and depression, are the most common comorbidities in both adults and pediatric populations, whereas attention deficit hyperactivity disorder is most common in children. The most common neurological comorbidities in patients with epilepsy are stroke and migraine, with migraine being more prevalent among younger patients while stroke being more common in older patients [22, 23]. Consistently, in this study, the most common neurological comorbidity was also stroke.

Our study suggests that the presence of comorbidities may decrease the probability of seizure freedom in senile epilepsy. Consistent with previous studies [8], here we found a trend toward lower seizure-freedom rate with increased number of comorbidities. However, the trend was not statistically significant after adjusting for confounding factors, which may be attributed to the small sample size in this study. Therefore, future studies with larger sample sizes are needed to explore the effects of comorbidities on the prognosis of epilepsy in the elderly.

In our study, the presence of neurological and psychiatric comorbidities was associated with a decreased probability of achieving seizure freedom. Neurological comorbidities, such as stroke, are more likely to result from structural changes in the brain, thus increasing the risk of recurrent seizures in elderly patients, possibly due to a causal effect. In addition, there may also be bidirectional effects. For example, dementia in people with epilepsy can lead to functional deterioration and behavioral changes, which, in turn, further aggravate the prognosis [24]. Previous studies have reported that stroke is the strongest independent predictor of acute symptomatic seizures and new seizures in people aged over 65 years [25, 26]. In the present study, depression (32/54) and anxiety (31/154) were the most common psychiatric comorbidities, which is consistent with previous studies [27–29]. Psychiatric comorbidities have negative impacts on the prognosis of epilepsy in the elderly population. We found a lower rate of seizure freedom in patients with anxiety and depression, which is consistent with previous reports [30]. Indeed, anxiety/depression and epilepsy may influence each other. The presence of psychiatric comorbidities will reduce the threshold of seizures and hinder the effective control of seizures, thus affecting the prognosis of epilepsy [31, 32]. Here, we found that patients with at least one bidirectional comorbidity had a lower rate of seizure freedom compared to those with no bidirectional comorbidity; moreover, the seizure-freedom rate decreased with increased number of bidirectional comorbidities. Therefore, psychiatric comorbidities should be addressed in each patient and properly managed to improve seizure control and the overall quality of life.

Our study has some limitations. First, this study was a retrospective study, thus there might be an information bias. The patient data were obtained primarily from the medical record system and by patient interview, which may have contributed to the missing of some information. Second, the sample size of this study was small, which may be a potential cause of the failure to detect statistical significance. Since the participants in this study were from only three tertiary hospitals in southwest China, there may be some selection bias. For example, patients from some less-developed regions, and those who were treated by general practitioners were not included. Therefore, the generalization of this result may be limited. Large-scale prospective cohort studies are needed to fully clarify the relationship between comorbidities and epilepsy outcomes in older adults.

## Conclusions

Our study revealed a high comorbidity rate and a low seizure-freedom rate among patients with senile epilepsy. Particularly, neuropsychiatric comorbidities can increase the risk of seizures and affect the survival rate in elderly patients with epilepsy. Our findings suggest that prevention and early treatment of comorbidities in patients with senile epilepsy may improve their outcomes and reduce the risk of death.

## Data Availability

All data generated or analyzed during this study are included in this article. Further enquiries can be directed to the corresponding author.

## Abbreviations

ASM: Anti-seizure medication
EEG: Electroencephalogram
HR: Hazard ratios
ILAE: The International League Against Epilepsy
OR: Odds ratio
